# Prognostic role of the advanced lung cancer inflammation index in cancer patients: a meta-analysis

**DOI:** 10.1186/s12957-019-1725-2

**Published:** 2019-11-02

**Authors:** Xin Hua, Jing Chen, Ying Wu, Jun Sha, Shuhua Han, Xiaoli Zhu

**Affiliations:** 1 Medical School of Southeast University, Nanjing, 210009 China; 2 Department of Respiratory, Zhongda Hospital of Southeast University, Dingjiaqiao 87, Gulou District, Nanjing, 210009 China

**Keywords:** Cancer, Meta-analysis, Advanced lung cancer inflammation index (ALI), Prognosis

## Abstract

**Background:**

Inflammation plays a critical role in the development and progression of cancers. The advanced lung cancer inflammation index (ALI) is thought to be able to reflect systemic inflammation better than current biomarkers. However, the prognostic significance of the ALI in various types of cancer remains unclear. Our meta-analysis aimed to comprehensively investigate the relationship between the ALI and oncologic outcomes to help physicians better assess the prognosis of cancer patients.

**Methods:**

The PubMed, Embase, Cochrane Library, China National Knowledge Infrastructure, and Wanfang databases were searched for relevant studies. Hazard ratios (HRs) with 95% confidence intervals (95% CIs) were calculated and pooled from the included studies. Furthermore, a sensitivity analysis was performed to evaluate the reliability of the articles. Finally, Begg’s test, Egger’s test, and the funnel plot were applied to assess the significance of publication bias.

**Results:**

In total, 1736 patients from nine studies were included in our meta-analysis. The median cutoff value for the ALI was 23.2 (range, 15.5–37.66) in the analyzed studies. The meta-analysis showed that there was a statistically significant relationship between a low ALI and worse overall survival (OS) in various types of cancer (HR = 1.70, 95% CI = 1.41–1.99, *P* < 0.001). Moreover, results from subgroup meta-analysis showed that the ALI had a significant prognostic value in non-small cell lung cancer, small cell lung cancer, colorectal cancer, head and neck squamous cell carcinoma, and diffuse large B cell lymphoma (*P* < 0.05 for all).

**Conclusions:**

These results showed that a low ALI was associated with poor OS in various types of cancer, and the ALI could act as an effective prognostic biomarker in cancer patients.

## Background

Cancer is one of the major causes of death worldwide [1]. In 2018, there were more than 18 million new cases diagnosed and 9.5 million cancer-related deaths [2]. Although great progress in treating cancer has been made over the past decade, the clinical outcome of cancer patients remains poor [3]. Therefore, identifying an effective prognostic index for patient survival could help clinicians adopt better preventive and therapeutic treatments, which could further reduce cancer mortality [4, 5].

Growing evidence indicates that cancer-related inflammation plays a critical role in the development and progression of various types of cancer [6–8]. At the early stages of tumorigenesis, various inflammatory cells and proinflammatory cytokines are activated, and these promote the formation of new blood vessels and lymphatic ducts, providing a tumor microenvironment beneficial to the growth and differentiation of tumor cells [9]. At later stages, cancer-related inflammation can destroy the function of immune cells, leading to a pro-metastatic environment [10–13]. Therefore, inflammatory markers are expected to be valuable prognostic biomarkers in cancer. For example, as a comprehensive index based on two blood factors, an increased neutrophil-to-lymphocyte ratio (NLR) is associated with a strong inflammatory response and a weak immune response, implying its effective prognostic value [14–16].

Cachexia in cancer patients is the result of the chronic systemic inflammatory response and often indicates a poor outcome for cancer patients [17, 18]. Sarcopenia is an important part of cancer cachexia syndrome and is associated with poor prognosis in multiple cancers, such as lung, gastrointestinal, and hepatopancreatobiliary malignancies [19, 20]. Previous studies have reported that the body mass index (BMI) has a close association with the sarcopenic status [21]. Serum albumin (ALB), which reflects the nutritional status, has also been proven to be associated with poor prognosis in many cancers [22–24]. A new inflammation-related marker, the advanced lung cancer inflammation index (ALI), was first determined to be an effective prognostic index in metastatic non-small cell lung cancer (NSCLC) [25]. The ALI combines the BMI, ALB, and the NLR (BMI × ALB/NLR). Therefore, the ALI has the potential to reflect systemic inflammation better than other biomarkers because it merges multiple nutritional and inflammatory indicators. Thus, it may have a better predictive value than other prognostic biomarkers in cancer patients.

However, a pooled study that analyzes the association between the ALI and clinical outcomes of patients with malignant diseases has not been systematically performed. Our meta-analysis aimed to explore the prognostic impact of the ALI in cancer patients, helping physicians predict clinical outcomes more effectively and easily and assisting them in the timely adjustment of therapeutic regimens, which further reduces mortality.

## Methods

### Search strategy

This study was performed according to the recommendations of the Preferred Reporting Items for Systematic Reviews and Meta-Analyses statement (Additional file 1). The PubMed, Embase, Cochrane Library, China National Knowledge Infrastructure, and Wanfang databases were searched for relevant studies without language, publication, or time restrictions (the publication period included database establishment to March 15, 2019). The following search terms were applied: “advanced lung cancer inflammation index” OR “ALI” OR “BMI x ALB / NLR” OR “BMI x serum albumin / NLR” OR “neutrophil-to-lymphocyte” AND “cancer” OR “tumor” OR “carcinoma.” Reference lists of the included articles were also scanned to identify potentially related studies.

### Inclusion and exclusion criteria

The following criteria were used for inclusion in this meta-analysis:

(i) Studies examining the association between the ALI and prognosis in patients with any type of cancer.
(ii) Sufficient data provided to calculate hazard ratios (HRs) and 95% confidence intervals (95% CIs) for the relationship between the ALI and overall survival (OS) in cancer patients.
(iii) The cutoff value of the ALI was clear.
(iv) If more than one article referred to the same population, only the study that included the most cases or the latest publication was included.

The following studies were excluded from the meta-analysis:

(i) Studies based on animal or cell experiments
(ii) Meta-analyses, reviews, case reports, or reports based on expert experience

### Data extraction and quality assessment

Two authors (HX and HSH) independently extracted the following data from all included studies:

(i) Basic information, including authors’ names, publication year, cancer type, country, study period, characteristics of the study population (sample size, age, and gender), survival type, treatments, clinical stage, cutoff value, cutoff selection, and study design
(ii) Statistical indicators, including HRs and corresponding 95% CIs for OS, from multivariate or univariate analysis or estimated from Kaplan-Meier survival curves using previously described methods if the HR could not be obtained directly [26]

The Newcastle-Ottawa Scale (NOS) was used to assess the quality of included studies, and a score ≥ 6 was considered an indicator of a high-quality study, whereas a score < 6 indicated a low-quality study [27]. Two reviewers (CJ and WY) independently evaluated the quality of the eligible studies, and all disagreements were resolved through discussion with a third author (ZXL).

### Statistical analysis

Stata software (version 12.0; Stata Corporation, College Station, TX, USA) was used to analyze the data in our study. HRs and 95% CIs were used to evaluate the association between the ALI and OS in cancer patients. A pooled HR > 1 was regarded as an indicator of poor prognosis in groups with a low ALI. The impact of the ALI on survival was considered statistically significant if the corresponding 95% CI for the summary HR did not overlap 1 unit. The Cochran’s *Q* test and *I*^2^ statistics were used to analyze heterogeneity between studies; *P* < 0.05 or *I*^2^ > 50% suggested significant heterogeneity among the included studies. If the homogeneity was significant, a random effects model was used. Otherwise, a fixed effects model was used [28]. Subgroup analyses were also performed on the basis of the median age, sample size, ethnicity, pathological type, clinical stage, treatment strategy, and ALI cutoff values. To explore the robustness of the overall statistical results, we performed a sensitivity analysis. Potential publication bias was assessed using Begg’s test, Egger’s test, and funnel plot. All *P* values were two-sided, and *P* < 0.05 was considered statistically significant.

## Results

### Study selection and characteristics

**Fig. 1:**
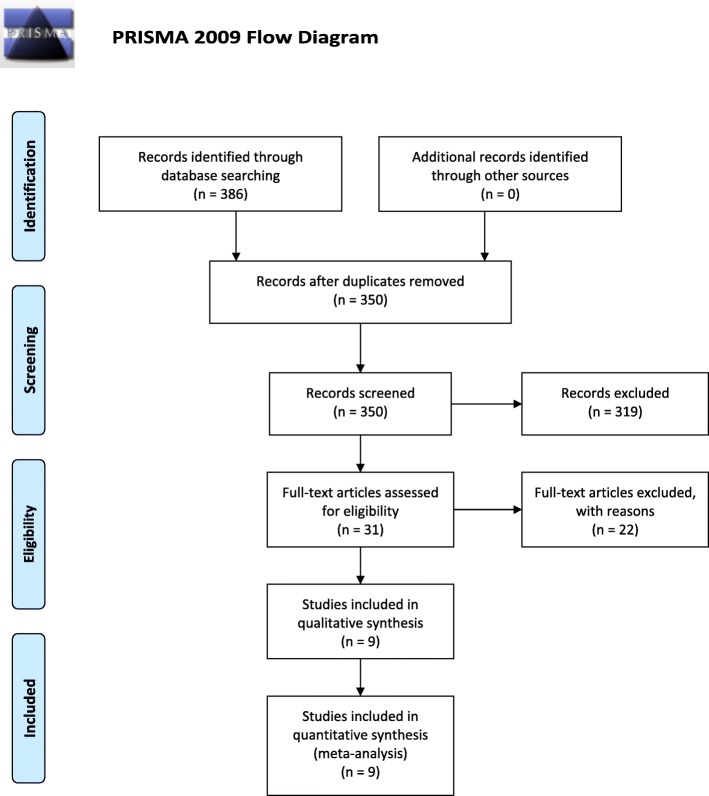
PRISMA flow diagram for this meta-analysis

The process of study selection is shown in Fig. 1. Ultimately, nine studies met our selection criteria; after excluding duplicated studies and reviewing the full texts of the manuscripts [25, 29–36], a total of 1736 cases were included. With respect to prognostic outcomes, nine studies reported OS, two studies reported progression-free survival, and one study reported disease-free survival. Among the included studies, four types of tumors were investigated, including lung cancer, colorectal cancer, head and neck squamous cell carcinoma, and diffuse large B cell lymphoma. The cutoff value of the ALI in the included studies ranged from 15.5 to 37.66, with a median of 23.2. The characteristics and demographic data of all included studies are presented in Table 1.

**Table 1:** Basic characteristics of the included studies

Study	Year	Cancer type	Country	Study type	Study period	No. of cases	Male patients, *n* (%)	Age, years	Survival type	Treatment	Stage	ALI cutoff value	Cutoff selection method
Jafri et al. [25]	2013	NSCLC	USA	R	2000–2011	173	116 (67%)	57 (34–88)	OS, PFS	No surgery	Metastatic	18.4	ROC curve analysis
He et al. [29]	2015	SCLC	China	R	2006–2011	365	310 (84.9%)	59 (22–82)	OS	No surgery	Mixed	19.50	Cutoff Finder
Kim et al. [30]	2016	SCLC	Korea	R	2010–2015	186	156(83.9%)	68.9 ± 9.4	OS	No surgery	Mixed	31.1	Cutoff value
Park et al. [31]	2017	Diffuse large B cell lymphoma	Korea	R	2006–2014	212	142 (67%)	59 (26–76)	OS, PFS	No surgery	Mixed	15.5	ROC curve analysis
Bacha et al. [32]	2017	NSCLC	France	R	2010–2012	41	41 (100%)	55 (41–76)	OS	No surgery	Metastatic	23.2	ROC curve analysis
Kobayashi et al. [33]	2018	NSCLC	Japan	R	2009–2014	166	74 (44.6%)	–	OS	Surgery	Non-metastatic	22.2	ROC curve analysis
Tomita et al. [34]	2018	NSCLC	Japan	R	2008–2012	341	173 (50.7%)	69 (median)	OS	Surgery	Non-metastatic	37.66	Cutoff Finder
Shibutani et al. [35]	2019	Colorectal cancer	Japan	R	2008–2016	159	87 (54.7%)	65 (18–89)	OS	No surgery	Metastatic	28.9	ROC curve analysis
Jank et al. [36]	2019	Head and neck squamous cell carcinoma	Austria	R	2002–2012	93	72 (77.4%)	58 (27–72)	OS, DFS	Surgery	Mixed	37.6	Cutoff value

*Abbreviations*: *NSCLC* non-small cell lung cancer, *SCLC* small cell lung cancer, *R* retrospective, *OS* overall survival, *PFS* progression-free survival, *DFS* disease-free survival, *Mixed* included patients with metastatic and non-metastatic disease, *ALI* advanced lung cancer inflammation index, *ROC* receiver operating characteristic curve

For quality assessment, the nine studies were evaluated using the NOS, and the scores were all ≥ 6, indicating that the included studies were all high-quality studies (Additional file 2).

### Relationship between the ALI and OS in various cancer types

**Fig. 2:**
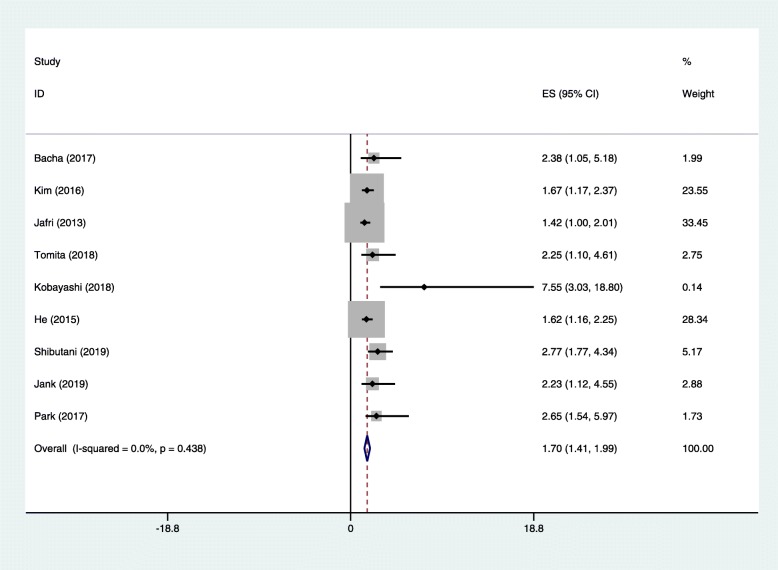
Forest plot of studies assessing the relationship between ALI and OS. Abbreviations: ALI advanced lung cancer inflammation index, OS overall survival

As shown in Fig. 2, there were nine studies with 1736 cases demonstrating the association between the ALI and OS in cancer patients. Our results indicated that a low ALI was significantly related to a poor outcome in cancer patients (HR = 1.70, 95% CI = 1.41–1.99, *P* < 0.001). Considering that heterogeneity was not obvious among the studies, a fixed effects model was applied.

In addition, subgroup analyses stratified by the median age, sample size, ethnicity, pathological type, clinical stage, treatments, and cutoff for ALI were also performed (Table 2). The results showed that a lower ALI was a significant predictive index of OS in NSCLC (HR = 1.55, 95% CI = 1.08–2.02, *P* < 0.001), small cell lung cancer (SCLC) (HR = 1.64, 95% CI = 1.24–2.05, *P* < 0.001), colorectal cancer (HR = 2.77, 95% CI = 1.77–4.34, *P* < 0.001), head and neck squamous cell carcinoma (HR = 2.23, 95% CI = 1.12–4.55, *P* = 0.011), and diffuse large B cell lymphoma (HR = 2.64, 95% CI = 1.54–5.97, *P* = 0.019). In terms of patient age and sample size, the ALI had a significant prognostic value for cancer patients regardless of the median patient age (≥ 60 years and < 60 years) or sample size (≥ 170 or < 170) (*P* < 0.001 for all). When studies were divided into those performed in Asian, North American, and European countries, the ALI was significantly related to OS only in studies from Asia and North America (*P* < 0.001 for both). When tumor stage was considered, the results showed that a lower ALI was a risk factor in patients with metastatic or mixed-stage tumors (*P* < 0.001 for both) but not in patients with non-metastatic disease. When performing subgroup analysis by treatment type, the association was still significant in patients who did not undergo surgery and those who underwent surgery (*P* < 0.001 for all). Furthermore, the ALI was indicated to be an effective prognostic factor when the cutoff for the ALI was > 23.2 and < 23.2 (*P* < 0.001 for all).

**Table 2:** Subgroup analysis of the relationship between the ALI and OS

Subgroup factor	Divided standard	No. of studies	HR (95% CI)	*P*	Heterogeneity	
					*I*^2^ (%)	*P*_het_
Median age (years)	< 60	5	1.59 (1.24–1.95)	< 0.001	0.0	0.66
	≥ 60	2	2.59 (1.56–3.63)	< 0.001	0.0	0.637
	Not reported	2	1.70 (1.11–2.30)	< 0.001	52.9	0.145
Sample size	< 170	4	2.61 (1.69–3.52)	< 0.001	0.0	0.614
	≥ 170	5	1.60 (1.29–1.90)	< 0.001	0.0	0.749
Ethnicity	Asian	6	1.80 (1.43–2.18)	< 0.001	12	0.34
	European	2	2.29 (0.97–3.61)	NS	0.0	0.91
	North American	1	1.42 (1.00–2.01)	< 0.001	–	–
Pathological type	NSCLC	4	1.55 (1.08–2.02)	< 0.001	19.2	0.29
	SCLC	2	1.64 (1.24–2.05)	< 0.001	0.0	0.898
	Colorectal cancer	1	2.77 (1.77–4.34)	< 0.001	–	–
	Head and neck squamous cell carcinoma	1	2.23 (1.12–4.55)	0.011	–	–
	Diffuse large B cell lymphoma	1	2.64 (1.54–5.97)	0.019	–	–
Clinical stage	Metastatic	3	1.64 (1.18–2.10)	< 0.001	52.7	0.12
	Non-metastatic	2	2.50 (0.78–4.21)	NS	0.0	0.20
	Mixed	4	1.70 (1.31–2.09)	< 0.001	0.0	0.76
Treatment	Surgery	3	2.37 (1.15–3.58)	< 0.001	0.0	0.43
	No surgery	6	1.66 (1.36–1.96)	< 0.001	0.5	0.41
Cutoff of ALI	< 23.2	4	1.55 (1.19–1.92)	< 0.001	13.7	0.32
	≥ 23.2	5	1.96 (1.47–2.44)	< 0.001	0.0	0.59

*Abbreviations*: *HR* hazard ratio, *95% CI* 95% confidence interval, *NSCLC* non-small cell lung cancer, *SCLC* small cell lung cancer, *Mixed* included patients with metastatic and non-metastatic disease, *ALI* advanced lung cancer inflammation index, *NS* not significant

### Sensitivity analysis and publication bias

**Fig. 3:**
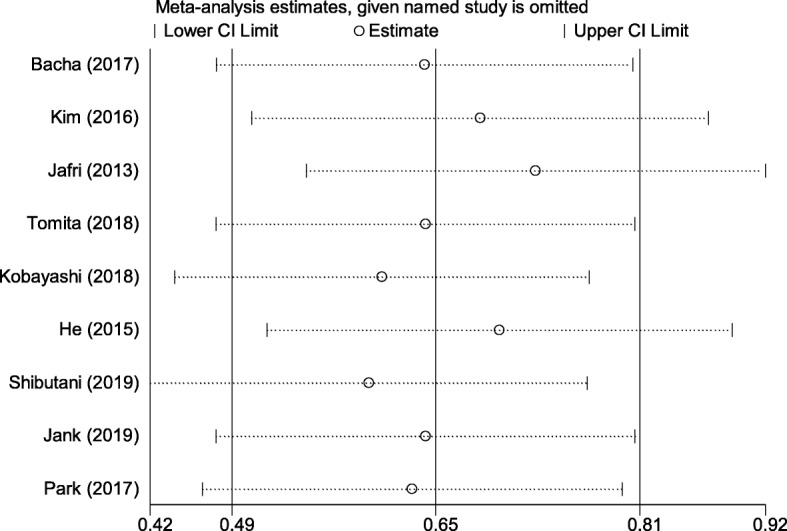
Sensitivity analysis of the relationship between the ALI and OS. Abbreviations: ALI advanced lung cancer inflammation index, OS overall survival

Sensitivity analysis was used to detect the robustness of these results, which showed that the pooled results were not altered by any one study, indicating that our conclusions are relatively reliable (Fig. 3).

**Fig. 4:**
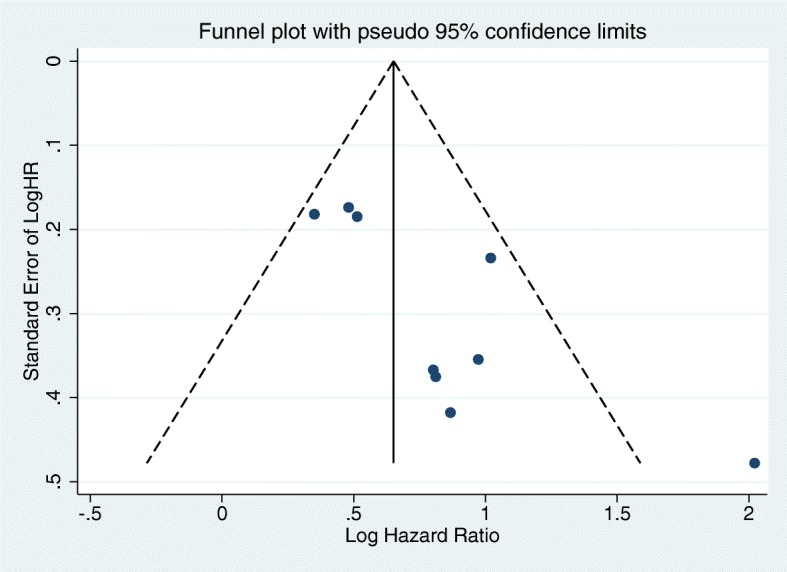
Funnel plot of the relationship between the ALI and OS. Abbreviations: ALI advanced lung cancer inflammation index, OS overall survival

Both Begg’s test (*P* = 0.048) and Egger’s test (*P* = 0.014) indicated that publication bias was present among the studies. An asymmetric funnel plot also proved this conclusion (Fig. 4).

## Discussion

A comprehensive search was conducted for published articles exploring the prognostic effect of the ALI on the survival outcomes of cancer patients. A total of 1736 cases from nine studies were included in our meta-analysis. The results of our study indicated that a low ALI was associated with worse prognosis (HR = 1.70, 95% CI = 1.41–1.99, *P* < 0.001). Furthermore, we observed consistent results in subgroups of various cancer types, including NSCLC, SCLC, colorectal cancer, head and neck squamous cell carcinoma, and diffuse large B cell lymphoma. In short, the ALI could act as a predictive factor for clinical outcomes in cancer patients.

Cancer progression is associated with a high level of systemic inflammation [37]. Many studies have shown that serum inflammatory markers, such as C-reactive protein (CRP) [38–40], the NLR [41–43], the platelet-to-lymphocyte ratio [44, 45], the Glasgow Prognostic Score (GPS) [46, 47], and the systemic immune-inflammation index [48, 49], are related to the clinical outcomes of cancer patients. Low body weight and hypoproteinemia are also both associated with persistent systemic inflammation [50–52], and the BMI and ALB have also been confirmed as effective prognostic markers for cancer patients [53, 54]. The ALI is an index developed on the basis of these current markers and could provide important prognostic information for cancer patients [55]. In addition, the ALI has been shown to be superior to other related inflammatory indicators used as predictive biomarkers in cancer. Kobayashi et al. examined the prognostic value of the ALI in lung adenocarcinoma patients and concluded that the ALI was an independent predictor of OS (HR = 7.55, 95% CI = 3.03–18.8) and had a better prognostic value than the NLR (HR = 3.91, 95% CI = 1.36–11.26) and GPS (HR = 1.24, 95% CI = 0.32–4.77) [33]. Tomita et al. revealed that the preoperative ALI and CRP levels were significant predictors of OS in patients with NSCLC and that the ALI (HR = 0.436, 95% CI = 0.278–0.679) was superior to the CRP level (HR = 0.631, 95% CI = 0.403–0.993) as a prognostic index [56]. The univariate analysis from Feng et al.’s study showed that the ALI, BMI, ALB, and NLR were significantly related to cancer-specific survival in esophageal squamous cell carcinoma patients [57]. However, the multivariate analysis demonstrated that only an ALI ≥ 18 was an independent prognostic factor of better cancer-specific survival (HR = 1.433, 95% CI = 1.048–1.959), but the NLR (HR = 1.436, 95% CI = 0.938–2.198), BMI (HR = 1.060, 95% CI = 0.752–1.494), and ALB (HR = 1.285, 95% CI = 0.905–1.824) were not. In summary, as a composite index combining the inflammatory state (NLR) and the nutritional state (BMI and ALB), the ALI may have a better discriminatory value than other biomarkers and remains a novel and effective inflammatory prognostic factor.

A subgroup analysis showed that, although the ALI had prognostic value in most subgroups, there was no difference in OS based on the ALI in European patients and in patients with non-metastatic disease. There are several possible reasons for these findings. First, the European subgroup contained a small number of studies (only two studies) and a small sample size. Second, the BMI, ALB, and NLR, which are components of the ALI, seem to have better prognostic value in advanced stages of cancer [58–60]. Therefore, the prognostic effect of the ALI on survival outcomes may be affected by the cancer stage. In the future, more data are needed in different stages of cancer to investigate the prognostic role of the ALI in different types of tumors, considering that the number of articles currently available is small.

Our study inevitably had some limitations. First, all of the studies included in this meta-analysis were retrospective, and the results may have thus been subject to potential bias. Second, confounding factors, such as the levels of tumor markers and history of chemoradiotherapy, might also affect the HR of the ALI in cancer patients; such an effect cannot be explored via subgroup analysis because the studies that were included did not provide sufficient information. Third, the cutoff value of the ALI was not uniform in different studies. Finally, publication bias existed in the studies that were included in our meta-analysis, which may be attributable to failure in publishing studies with negative results or with other variables.

## Conclusions

In summary, our study revealed that a low ALI was significantly correlated with worse OS in cancer patients. Therefore, the ALI could be a reliable predictor for prognosis in cancer patients, providing consistent results for different cancer types. In the future, more large-scale, prospective, well-designed studies are needed to verify the association of the cutoff values of the ALI and tumor stage with the prognostic features of the ALI for patients with different types of cancer.

## Supplementary information

**Additional file 1.** PRISMA 2009 checklist used in this meta-analysis.

**Additional file 2.** Newcastle-Ottawa Scale for quality assessment.

## Data Availability

All data generated or analyzed during this study are included in this published article [and its supplementary information files].

## Abbreviations

95% CI: 95% confidence interval
ALB: Serum albumin
ALI: Advanced lung cancer inflammation index
BMI: Body mass index
CRP: C-reactive protein
GPS: Glasgow Prognostic Score
HR: Hazard ratio
NLR: Neutrophil-to-lymphocyte ratio
NOS: Newcastle-Ottawa Scale
NSCLC: Non-small cell lung cancer
OS: Overall survival
SCLC: Small cell lung cancer
