# Supplementary material for: Prognostic role of the advanced lung cancer inflammation index in cancer patients: a meta-analysis
Source: World J Surg Oncol. 2019 Nov 2;17:177. doi: 10.1186/s12957-019-1725-2 (PMC6825711; doi:10.1186/s12957-019-1725-2)
Supplement: Supplementary file 2 — Additional file 2. Newcastle-Ottawa Scale for quality assessment. [file 12957_2019_1725_MOESM2_ESM.doc]

**Additional file 2: Newcastle-Ottawa Scale for quality assessment**

| **Author, year** | **Selection** | | | | **Comparability** | **Outcome** | | | **Total score** |
| --- | --- | --- | --- | --- | --- | --- | --- | --- | --- |
|  | **Exposed cohort** | **Non-exposed cohort** | **Ascertainment of exposure** | **Outcome of interest** | **Control for factor** | **Assessment of outcome** | **Sufficient duration of follow-up** | **Adequacy of follow-up** |  |
| Jafri et al [25] | * | * | * | * | ** | * |  | * | 8 |
| He et al [29] | * | * | * | * | * | * | * | * | 8 |
| Kim et al [30] | * | * | * | * | * | * | * | * | 8 |
| Park et al [31] | * | * | * | * | * | * |  | * | 7 |
| Bacha et al [32] | * | * | * | * | * | * |  | * | 7 |
| Kobayashi et al [33] | * | * | * | * | ** | * | * | * | 9 |
| Tomita et al [34] | * | * | * | * | * | * |  |  | 6 |
| Shibutani et al [35] | * | * | * | * | ** | * |  | * | 8 |
| Jank et al [36] | * | * | * | * | ** | * | * |  | 8 |

**Abbreviations:**

*The article scored one point in this area

**The article scored two points in this area
